# Tubal Pregnancy

**Published:** 1914-08

**Authors:** 


**Tubal Pregnancy.** Wightman of Omaha, Western Med. Rev., May, 1914, reports a case in which the positive result of the Abderhalden test was of great value in the diagnosis. Operation, removal of clots, tube and ovary, successful.

